# Toxicity of Vanadium during Development of Sea Urchin Embryos: Bioaccumulation, Calcium Depletion, ERK Modulation and Cell-Selective Apoptosis

**DOI:** 10.3390/ijms23116239

**Published:** 2022-06-02

**Authors:** Roberto Chiarelli, Rosaria Scudiero, Valeria Memoli, Maria Carmela Roccheri, Chiara Martino

**Affiliations:** 1 Department of Biological, Chemical and Pharmaceutical Sciences and Technologies (STEBICEF), University of Palermo, Viale delle Scienze Building 16, 90128 Palermo, Italy; maria.roccheri@unipa.it (M.C.R.); chiaracomlib@yahoo.it (C.M.); 2 Department of Biology, University Federico II, 80126 Napoli, Italy; rosaria.scudiero@unina.it (R.S.); valeria.memoli@unina.it (V.M.)

**Keywords:** vanadium bioaccumulation, calcium uptake, *Paracentrotus lividus* embryos, stress, cell-selective apoptosis

## Abstract

Vanadium toxicology is a topic of considerable importance as this metal is widely used in industrial and biomedical fields. However, it represents a potential emerging environmental pollutant because wastewater treatment plants do not adequately remove metal compounds that are subsequently released into the environment. Vanadium applications are limited due to its toxicity, so it is urgent to define this aspect. This metal is associated with sea urchin embryo toxicity as it perturbs embryogenesis and skeletogenesis, triggering several stress responses. Here we investigated its bioaccumulation and the correlation with cellular and molecular developmental pathways. We used cytotoxic concentrations of 1 mM and 500 μM to perform quantitative analyses, showing that vanadium accumulation interferes with calcium uptake during sea urchin development and provokes a disruption in the biomineralization process. At the end of the whole treatment, the accumulation of vanadium was about 14 and 8 μg for embryos treated respectively with 1 mM and 500 μM, showing a dose-dependent response. Then, we monitored the cell signaling perturbation, analyzing key molecular markers of cell survival/cell death mechanisms and the DNA fragmentation associated with apoptosis. This paper clarifies vanadium’s trend to accumulate directly into embryonic cells, interfering with calcium uptake. In addition, our results indicate that vanadium can modulate the ERK pathway and activate a cell-selective apoptosis. These results endorse the sea urchin embryo as an adequate experimental model to study metal-related cellular/molecular responses.

## 1. Introduction

The accumulation of metals in aquatic organisms can occur directly from polluted water or indirectly from contaminated feed sources [1]. Among the several pollutants that can contribute to aquatic pollution, metals and pharmaceuticals are amongst the most widespread and serious dangers for the aquatic environment [2,3,4,5,6,7].

Vanadium (V), a metal that occurs from natural and industrial sources (such as industries producing the metal, its chemical compounds, alloys, and other products), has recently received great attention for its toxicity, that usually increases in parallel with its oxidation state (+2, +3, +4, +5) [8].

V compounds represent an encouraging search field in medicine as several studies have revealed the potential role of V-based products for the treatment of several pathologies (dysmetabolic syndromes, neurodegenerative diseases and heart disorders) [9,10].

V compounds could have several biological applications, ranging from apoptosis, cell cycle arrest, interference with ions transport system, inhibition of mRNA synthesis, cell morphology changes, changes in metabolic pathways, phosphorylase enzyme inhibition and cell signaling, to formation of reactive oxygen species, lipid peroxidation, inhibition of viral mRNA polymerase, inhibition of virus binding to the host cell, penetration and interaction with virus protein cages [11].

Interesting effects were observed on the interaction of V with proteins and/or enzymes such as tyrosine protein phosphatases, P-type ATPases, RNA triphosphatases, myosin and actin [12].

These experimental data could lead researchers to use V in the composition of new metallodrugs, as has already happened for some metals now used for medical diagnosis: iodine (I), barium (Ba) and gadolinium (Gd) [13,14,15].

However, a serious problem associated with the use of these compounds is that pharmaceutical residues (or drugs) are not adequately removed by wastewater treatment plants, becoming emerging pollutants of aquatic environments. These pollutants can produce toxic effects for humans and aquatic organisms, which has turned research studies onto their fundamental effects [5]. A similar scenario has been observed for Gd, an element used in magnetic resonance imaging that is now considered dangerous for the environment [16], and it can induce severe negative effects on aquatic organisms as it was demonstrated for sea urchin embryos [14,17].

The sea urchin embryo *Paracentrotus lividus* (Lamarck, 1816) is traditionally considered an excellent animal system in ecotoxicological studies because it directly interacts with the aquatic environment and actively responds to the presence of contaminants activating specific cellular and molecular defense mechanisms [18,19,20,21].

The sea urchin embryonic skeleton, a calcareous structure composed of magnesium calcite, represents a good marker in ecotoxicology because of its high sensitivity to different environmental stressors [19,22]. Embryo exposure to various metals commonly detected in the aquatic environment (cadmium (Cd), manganese (Mn) and Gd) strongly perturbs the biomineralization process, most likely because these elements including calcium (Ca) compete with each other across Ca channels during their uptake from seawater [23,24,25].

Recently, we reported a toxicological summary of the responses activated by V on *P. lividus* sea urchin embryos, highlighting the consequences related to several V concentrations administered from fertilization to the pluteus stage. We observed altered embryo phenotypes, skeletal absence or malformations, cellular stress response mediated by HSPs, autophagy and apoptosis and a modulation of metal-related proteolytic activities [26,27].

In this work, we extended our studies about V-toxicity to determine its possible bioaccumulation, and to obtain functional information on high V-exposure effects. With this aim, we used two different V concentrations (1 mM and 500 μM) and, through different approaches, we correlated the kinetics of V-accumulation with the level of Ca uptake during development. Then, we monitored the cellular perturbation, analyzing apoptotic cell death and molecular markers related to this process.

This paper clarifies the trend of V to accumulate directly into embryonic cells, interfering with the Ca uptake. Data suggest a modulation of extracellular signal-regulated kinase (ERK) activation and an activation of cell-selective apoptosis.

Our data provide new insight into sea urchin development under V-exposure conditions and the ability of these embryos to activate a specific response.

## 2. Results

### 2.1. V-Bioaccumulation Competes with Ca Uptake

**Figure 1:**
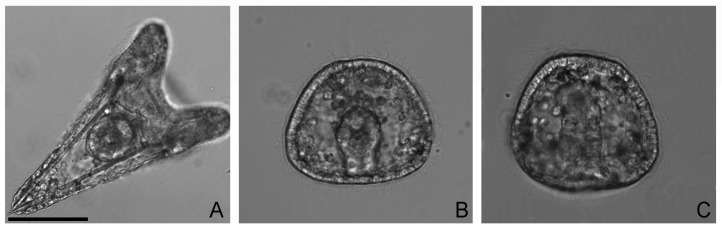
Pictures of representative embryos at 36 h of development/treatment. Control embryo (**A**), 1 mM V-treated embryo (**B**), 500 μM V-treated embryo (**C**). Bar: 100 μm.

We inspected the effects of V exposure in 36 h *P. lividus* embryos, when controls were plutei displaying a normal skeletal structure (Figure 1A). According to our previous studies, high V concentrations (1 mM or 500 μM) dramatically impair skeletogenesis (Figure 1B,C) as no spicules were present.

To study a possible connection between skeleton elongation and Ca uptake in V-exposed embryos, we measured the absorption levels of these metals during treatment. *P. lividus* embryos were constantly grown from fertilization in 1 mM or 500 μM V for a time comprised from 12 to 42 h. V and Ca content was tested by ICP-MS, calculating the concentration of metal in about 250,000 embryos.

**Figure 2:**
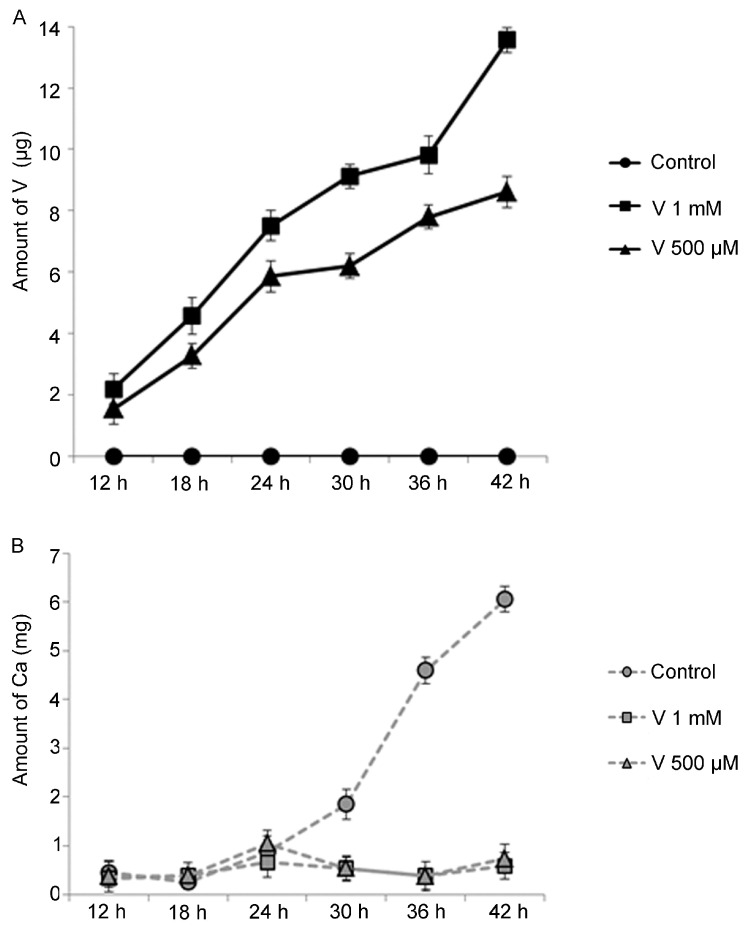
Amount of V and Ca incorporated during the time after 12, 18, 24, 30, 36 and 42 h of development/treatment. Embryos were cultured in 1 mM or 500 μM of V. V (**A**) and Ca (**B**) content were detected by Inductively Coupled Plasma Mass Spectrometry (ICP-MS), determining the metal quantity in about 250,000 embryos. Experiments were performed in triplicate and data are expressed as means ± standard deviation (*n* = 3 ± SD).

The analyses demonstrated that V accumulated into embryos during time, for both tested concentrations (Figure 2A). Embryos incorporated a comparable level of V for both concentrations until the hatched blastula stage, at 12 h of development. After 18, 24, 30, 36 and 42 h, V speedily accumulated in a dose- and time-dependent manner, while it was always undetectable in controls (Figure 2A). Interestingly, an inverse trend was recorded for Ca levels in V-exposed embryos (Figure 2B). A weak increase in Ca levels was detected at 24 h of treatment, representing in control embryos the developmental stage in which Ca uptake increases physiologically. Then, the level of Ca decreased for both V concentrations and a weak increase was observed only at 42 h of treatment.

Unlike in treated embryos, Ca rapidly accumulated during physiological development in controls, starting from the gastrula stage (24 h of embryogenesis). Ca had a first increase at prism stage (30 h) and then markedly increased at the pluteus stage (36 and 42 h), in parallel to the elongation of the skeleton. In particular, the level of measured Ca was proportional to the skeleton mass and spicule elongation as described in previous papers [24,26,28].

We tested the correspondence of nominal vs. analytical V concentrations in the initial embryonic culture medium (time zero) and during the exposure time through two check points (24 h and 42 h). At time zero, the analytical concentration values corresponding to the 1 mM and 500 μM were respectively 62 and 35.4 mg/L. After 24 h of exposure, V values in the 1 mM and 500 μM culture mediums were 59.3 and 29.4 mg/L, respectively. After 42 h of exposure, the V values in the 1 mM and 500 μM culture mediums were 64.5 and 27.2 mg/L, respectively.

### 2.2. ERK Modulation on Embryos Exposed to V

**Figure 3:**
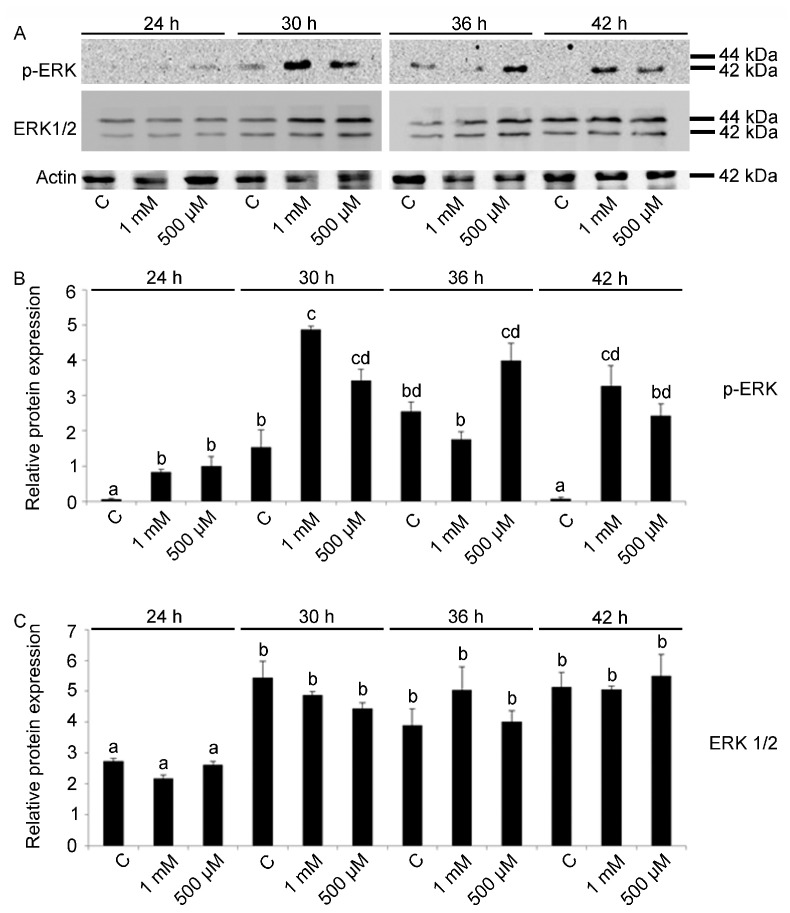
Immunoblotting detection and quantitative analysis for pERK and ERK 1/2. (**A**) Total lysates of control and V-treated (1 mM, 500 μM) embryos after 24, 30, 36 and 42 h of development/treatment. Actin was used as a loading control. Histograms show the densitometric analysis of bands identified for (**B**) pERK and (**C**) ERK 1/2. Relative protein expression, reported as arbitrary units, was calculated as the band density ratio to that of actin. Experiments were performed in triplicate and data are expressed as means ± standard deviation (*n* = 3 ± SD). Data were analyzed by one-way ANOVA. Treatments with the same lowercase letter do not differ (Tukey HSD).

Next, considering that skeletogenesis is regulated by PMC through ERK-mediated signaling pathway [29,30,31], we investigated a possible modulation of ERK on V-exposed embryos.

p-ERK levels were found to be related to V concentration (F11,24: 17.72; *p* < 0.0001). In particular, for exposure to the highest V-dose (1 mM), the activation of ERK starts at 24 h of treatment and a strong activation occurs after 30 and 42 h (Tukey’s HSD: 24 h 1 mM = 36 h 1 mM < 30 h 1 mM = 42 1 mM). At these developmental stages, massive apoptotic processes were activated [26]. In embryos treated with V 500 μM, a parallel increase was observed after 24 h of treatment and a more marked signal was observed after 30 h of development (Figure 3A,B).

In control embryos, the level of p-ERK was low at 24 and 42 h, with an increase at early pluteus stage at 30 and 36 h (Tukey’s HSD: C 24 h = C 42 h < C 30 h = C 36 h).

V treatment did not affect ERK protein expression, as a significant difference was found only in embryos grown at 24 h with or without V when compared to embryos at later developmental stages (Tukey’s HSD: C 24 h < C 30 h, C 36 h, C 42 h). Here, unlike what was reported in the literature, concerning the difficulty of discriminating ERK 1 and ERK 2 in sea urchin embryos [24,31], the antibody used was able to recognize both bands (Figure 3A,C).

### 2.3. Cell Cycle Arrest and Apoptosis

It is known that activated ERK is also involved in survival or cell death pathways, depending on the inducing agents and the cell type [32]. To identify a possible relationship between the level of activated ERK and programmed cell death mechanisms, we studied the level of CHOP 10/GADD 153, a factor related to cell cycle arrest in G1-S phase and programmed cell death [33].

**Figure 4:**
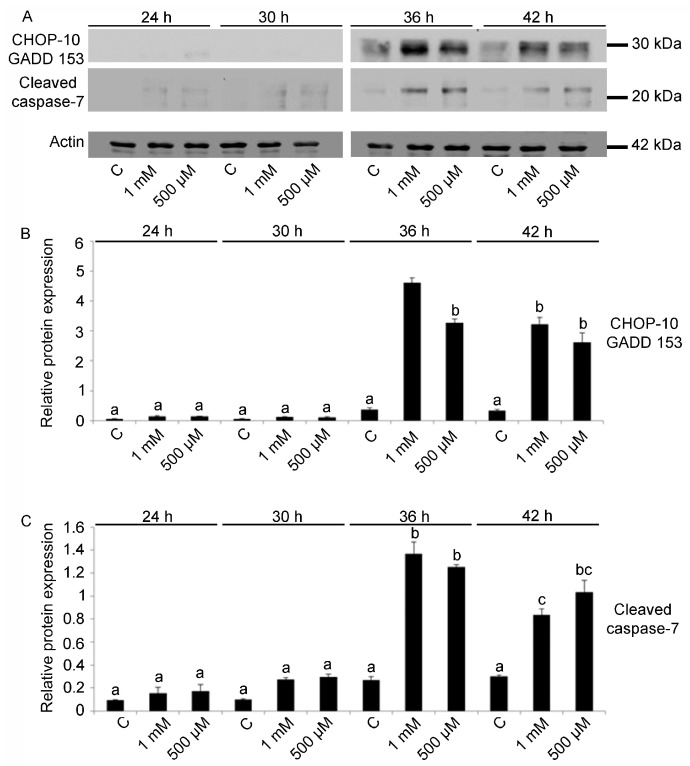
Immunoblotting detection and quantitative analysis for CHOP 10/GADD 153 and cleaved caspase 7. (**A**) Total lysates of control and V-treated (1 mM, 500 μM) embryos after 24, 30, 36 and 42 h of development/treatment. Actin was used as a loading control. Histograms show the densitometric analysis of bands identified for (**B**) CHOP-10/GADD 153 and (**C**) cleaved caspase 7. Relative protein expression, reported as arbitrary units, was calculated as the band density ratio to that of actin. Experiments were performed in triplicate and data are expressed as means ± standard deviation (*n* = 3 ± SD). Data were analyzed by one-way ANOVA. Treatments with the same lowercase letter do not differ (Tukey HSD).

V treatment affected CHOP 10/GADD 153 protein levels (F11,24: 120.92; *p* < 0.0001). In V-exposed embryos, for both concentrations, significantly high levels were detected at 36 and 42 h of development (Tukey’s HSD: C 36 h = C 42 h < 36 h V 500 μM = 42 h V 500 μM = 42 h V 1 mM < 36 h V 1 mM) (Figure 4A,B).

These results related to cell cycle arrest have been associated with the levels of the cleaved caspase 7 protein, as V-exposure had a significant effect on its expression (F11,24: 61.54; *p* < 0.0005). In V-exposed embryos, the activation of cleaved caspase 7 starts at 36 h and decreases at 42 h (Tukey’s HSD: C 36 h < 36 h V 500 μM = 36 h 1 mM; C 42 h < 42 h V 500 μM = 42 h V 1 mM) (Figure 4A,C). No differences in the cleaved caspase 7 levels were detected among controls at all developmental stages (Tukey’s HSD: C 24 h = C 30 h = C 36 h = C 42 h).

### 2.4. DNA Fragmentation and Evidence of a Cell-Selective Apoptosis

**Figure 5:**
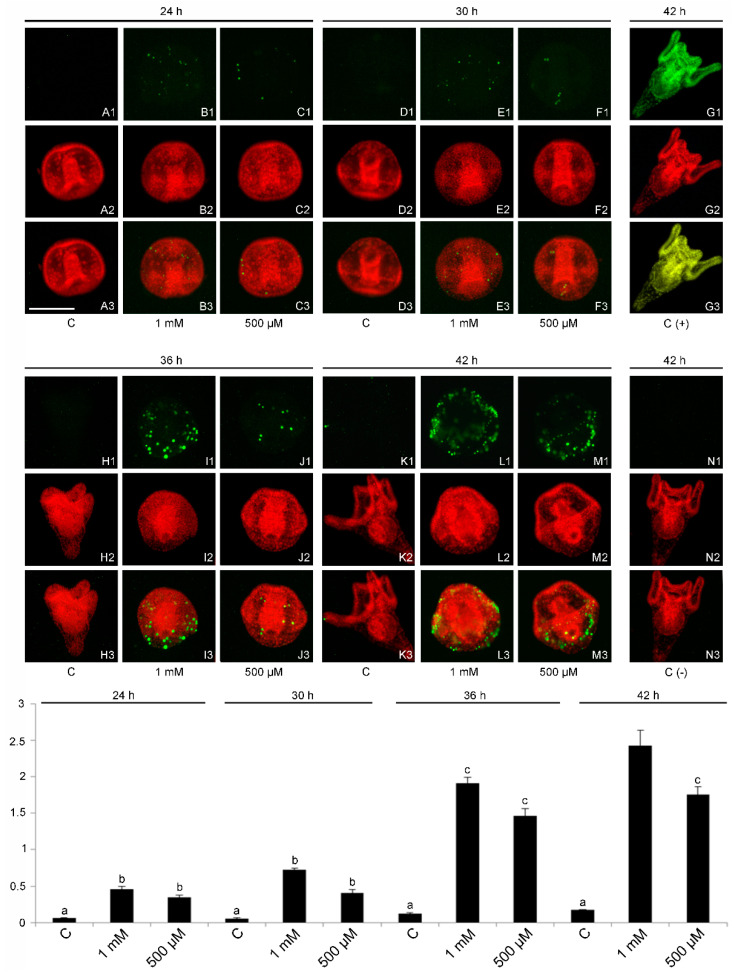
Fluorescent TUNEL assay and densitometric analysis. Pictures of demonstrative embryos at 24, 30, 36 and 42 h of development/treatment. DNA fragmentation (**A1**–**N1**). Nuclei marked with propidium iodide (**A2**–**N2**). Merge of both signals (**A3**–**N3**). Control embryos (**A1**–**A3**,**D1**–**D3**,**H1**–**H3**,**K1**–**K3**); 1 mM V-treated embryos (**B1**–**B3**,**E1**–**E3**,**I1**–**I3**,**L1**–**L3**); 500 μM V-treated embryos (**C1**–**C3**,**F1**–**F3**,**J1**–**J3**,**M1**–**M3**). Positive control embryo at 42 h of development (**G1**–**G3**). Negative control embryo at 42 h of development (**N1**–**N3**). Bar = 100 μm. Histograms showing data related to the quantitative analysis of fluorescence from apoptotic DNA. Experiments were performed in triplicate and data are expressed as means ± standard deviation (*n* = 3 ± SD). Data were analyzed by one-way ANOVA. Treatments with the same lowercase letter do not differ (Tukey HSD).

To evaluate a possible DNA damage induced by V and to confirm the cleaved caspase 7 results, a TdT assay was carried out (Figure 5A–N). This analysis was also applied in an attempt to establish whether there are cells that are more damaged than others. The TdT assay showed the apoptotic DNA fragmentation, in relationship to the V dose and exposure time (F11,24: 80.68; *p* < 0.0001). While no massive fragmentation was observed in control embryos at all developmental stages, significantly high levels were observed in V-treated embryos. In V-exposed embryos, apoptotic nuclei started to be detectable at 24 h of development for both V concentrations, when control embryos were at the gastrula stage (Tukey’s HSD: C 24 h < 24 h V 500 μM = 24 h V 1 mM). Only some cells showed nuclei with apoptotic DNA fragmentation and in particular, in many embryos, only the nuclei of the ectodermal cells were observed.

After 30 h of exposure time, when controls were prisms, the number of cells with apoptotic DNA fragmentation was increased and their localization confirmed that only the cells of the external layer of the embryo were involved (Tukey’s HSD: C 30 h < 30 h V 500 μM = 30 h V 1 mM).

At 36 h of exposure, when controls were early plutei, the level of apoptotic DNA fragmentation was further increased (Tukey’s HSD: C 36 h < 36 h V 500 μM = 36 h V 1 mM). Regarding their localization, the affected cells seemed to be only ectodermic, although at this stage of development, especially for embryos treated with the highest V concentration, there were severe morphological anomalies that perturbed the correct localization of cells.

At 42 h of development, the level of apoptotic DNA fragmentation increased in 1 mM V-exposed embryos (Tukey’s HSD: C 42 h < 42 h V 500 μM < 42 h V 1 mM), with a greater involvement of the ectodermal cells. At overall developmental times, a relationship between the level of apoptotic DNA fragmentation and the exposure time was observed (Tukey’s HSD: 24 h V 500 μM = 24 h V 1 mM = 30 h V 500 μM = 30 h V 1 mM < 36 h V 500 μM = 36 h V 1 mM = 42 h V 500 μM < 42 h V 1 mM). In particular, at 42 h we also observed a dose-dependent relationship with the V concentration. The type of activated apoptosis, based on the partial number of embryonic cells involved, was selective, differentiating itself from total apoptosis which is generally triggered by other metals (e.g., Cd). Few apoptotic DNA nuclei were observed in control plutei (Figure 5K1) representing basal physiological apoptosis (42 h of development), especially in the apical-, pre- and post-oral arms [14,26,34,35,36,37].

## 3. Discussion

V is an element having multiple possible roles in animals and human. Although toxic effects induced by high doses have been reported, many studies suggested the opportunity of exploiting V-based products as metalpharmaceuticals [9]. It is well known that metals released in the environment, in a close contact with organisms, can accumulate along the food chain. In a similar way, V-based products may pose a threat for marine organisms [5].

Embryogenesis and skeleton growth are sensitive aspects to estimate the impact of environmental alterations during sea urchins development [14]. During biomineralization, embryos employ Ca and Mg obtained from seawater to construct the skeleton [22]. The presence of polluting metals perturbs this fragile process due to competition for the ingression and storage of Ca in the cells, as we previously reported for some metals (i.e., Cd, Mn, Gd) that are able to compete with Ca uptake [23,24,25,38]. Our previous work reported, for the first time, an integrative and comparative analysis of the toxic effects induced by V on sea urchin embryos [26]. The exposure to different V-concentrations (ranging from those detected in moderately/non-polluted seawater to very cytotoxic doses) induced, after 36 h of treatment, morphological and molecular responses: altered embryogenesis, missing or malformed skeleton and an increased expression of heat shock proteins, autophagy and apoptosis [26].

From our previous data on the morphological and skeletal analysis, a possible competition between V and Ca was hypothesized.

ICP-MS data in embryos confirmed that V accumulates continuously as development proceeds, proportionally to its concentration. Quantitative investigations carried out on the embryo culture mediums, to establish the correspondence between analytical and nominal concentration, showed that for the entire exposure time (0–42 h), the conditions of the embryo culture us allowed to maintain constant the V concentration.

The accumulation kinetics always increased, even considering the activation of pathways that may be involved in cell death (p-ERK activation), cell cycle blocking (CHOP 10/GADD 153) and the activation of apoptotic processes (cleaved caspase 7, DNA fragmentation). This suggests that the metal is not excreted by embryonic cells but accumulates even if the apoptotic processes were simultaneously activated. This condition could be explained through data obtained from the whole-mount analysis, which reconstructed an image of the whole embryo in situ. Embryonic cells were partially involved in nuclear apoptotic fragmentation (detected by TdT assay), evidencing this phenomenon mainly into ectodermal cells. The mesodermal and endodermal cells did not appear to be involved in this process of cell-selective apoptosis and this would justify the continuous accumulation kinetics of V in embryos. In particular, microscopic fluorescence inspections indicated that PMCs were not involved in apoptotic processes.

However, the inability of these cells to carry on skeletogenesis could be due to the limited bioavailability of endogenous Ca, which was markedly reduced in all V-treated embryos. The Ca drop (determined by ICP-MS) occurring during development indicates that V affects Ca uptake and, subsequently, interferes with biomineralization. Considering that the swimming and feeding abilities of sea urchin larvae are directly determined by arm length [39], ultimately affecting their developmental success, V exposure would potentially reduce their survival in nature.

The role of the ERK signaling pathway in sea urchin embryos has been previously described [31,40], showing that its inhibition induces altered embryogenesis and abnormal skeletogenesis [31]. In addition, the ERK pathway depends on several modulators and Ca is one of the most important [41]. We found a peak of ERK activation for each V treatment. In particular, embryos treated with 1 mM showed a peak of ERK activation after 30 h of treatment when the amount of accumulated V was 70% of the whole treatment; embryos treated with 500 μM showed a peak of ERK activation after 36 h of treatment, when the amount of accumulated V was 90%. In both cases the amount of Ca content was 12 times lower than in the control embryos. It has been demonstrated that the MAPK pathway is perturbed in sea urchin eggs when associated with an artificially altered Ca content [42]. This would explain the results reported, here since the modulation of ERK in sea urchin embryos may depend on the Ca content, similar to what happens in eggs.

In addition, ERK activation could have a link with programmed cell death. The ERK (Ras/Raf/extracellular signal-regulated kinase) signaling pathway has an important role in all cell functions and consequently needs a specific control of its spatial and temporal activity [32]. Based on different stimuli, ERK activity mediates several antiproliferative events, such as the promotion of permanent cell cycle arrest and apoptosis [43]. We observed a marked signal of cell cycle arrest in G1-S phase for both V treatments starting from the early pluteus stage (as indicated by signals of CHOP 10/GADD 153 protein), as well as apoptosis (as indicated by cleaved caspase 7 signals and in situ analysis for apoptotic DNA fragmentation). These data correlate with the quantitative investigations conducted by ICP-MS for V and Ca and the activation levels of ERK, since high V bioaccumulation matches with the Ca drop and the level of ERK phosphorylation.

Finally, we can speculate that the effects of V on skeletogenesis are due to its bioaccumulation and consequent Ca depletion. The latter also plays a crucial role in the physiology and biochemistry of embryonic cells. Ca ions play a vital role in the pathways of signal transduction, because it works as a second messenger, and many enzymes require Ca as cofactor.

Our data, obtained using V, add some information on the type of response that sea urchin embryos activate in response to metals. The type of response is adequately based on the extent of the damage. Based on our previously studies, the most privileged pathways activated in response to metal stress are represented by heat shock proteins, autophagy and apoptosis; the latter appears to have greater response variation in sea urchin embryos. Indeed, Cd activates an apoptosis that affects the totality of embryonic cells; Mn does not activate an apoptotic response and Gd activates a cell-selective apoptotic response only if combined with temperature increase [14,24,26,35,36,44]. On the contrary, V activates a cell-selective apoptosis, even at concentrations similar to those of the polluted sites.

## 4. Materials and Methods

### 4.1. Embryos Cultures and V-Exposure

Adult specimens of sea urchins (*P. lividus*) were harvested from the Mediterranean Sea, in the Favignana Island MPA (Marine Protected Area), Sicily. Embryos were developed as described by Chiarelli et al. [26], according to the standard procedures applied in toxicology and in studies about metals exposure in sea urchin embryo cultures [35,45]. V-exposure was performed by exposing embryos in 1 mM or 500 μM of sodium orthovanadate (Na_3_VO_4_, thereafter V) (Sigma-Aldrich, St. Louis, MO, USA, cod. S6508), from fertilization until the pluteus stage. We used Na_3_VO_4_ since the V oxidation state in this compound (+5) is the most common form present in physiological circumstances [46]. To ensure the presence of vanadate monomers, the solution was boiled until translucent and the pH was readjusted to 10. Before boiling the solution appeared yellow/orange due to decavanadate presence. The absence of decavanadate in the final solution was confirmed by reading the stock solution (1:100 diluted) from 220 to 700 nm, by a Cary 100 UV-Visible Spectrophotometer, as described by Chiarelli et al. [27].

### 4.2. V and Ca Quantitative Analysis

50 mL of embryo cultures (control or V-exposed embryos at 12, 18, 24, 30, 36, 42 h of development) were collected by centrifugation at room temperature (800 rpm, 5 min) and washed three times. Embryo pellets were weighted and stored at −20 °C until use. V concentration was measured by digesting the samples with hydrofluoric acid (50%) and nitric acid (65%) at a ratio of 1:2 (*v*:*v*) in a microwave oven (Milestone-Digestion/Drying Module mls 1200, Milestone srl, BG, Italy). The concentration of each element in the digested samples was measured by Inductively Coupled Plasma Mass Spectrometry (ICP-MS Aurora M90, Bruker, Billerica, MA, USA). Accuracy of element measurements was checked by the concurrent analysis of standard reference material.

The correspondence of nominal vs. analytical concentrations used in this study was determined by a set of ICP-MS analyses using an Aurora M90 apparatus (Bruker, Billerica, MA, USA).

Experiments were performed in triplicate and data are expressed as means ± standard deviation (*n* = 3 ± SD).

### 4.3. Electrophoretic Analysis and Immunoblotting

Pellets were recovered from 10 mL of embryo cultures at 24, 30, 36 and 42 h of development and were homogenized using a lysis buffer (7 M CH_4_N_2_O, 2% CHAPS, 10 mM C_4_H_10_O_2_S_2_) containing a mix of protease inhibitors with broad specificity for the inhibition of serine, cysteine, aspartic proteases and aminopeptidases (Sigma-Aldrich, St. Louis, MO, USA, cod. P8340).

The Bradford method was used to determinate the protein concentration and 30 μg of extracted proteins was separated by 10% SDS-PAGE.

After electrophoresis, protein bands were transferred to 0.45 μm nitrocellulose blotting membranes and reacted with the following primary antibodies: ERK 1 + ERK 2 (Abcam, Cambridge, UK, cod. ab17942); p-ERK (Santa Cruz Biotechnology, Delaware, CA, USA, cod. sc-7383); cleaved caspase 7 (Asp198) (Cell Signaling Technology, Danvers, MA, USA, cod. 9491); GADD 153 (Santa Cruz Biotecnology, Delaware, CA, USA, cod. sc-793); and actin (Sigma-Aldrich, St. Louis, MO, USA, cod. A5060), according to the following dilution: 1:200; 1:500; 1:500; 1:300; 1:500, in 5% Blotto non-fat milk/TBS-T. The used secondary antibodies were anti-rabbit IgG (GE Healthcare, Chicago, IL, USA, cod. NA934) or anti-mouse IgG (GE Healthcare, Chicago, IL, USA, cod. NA931), dilution: 1:2500.

Protein immunoreactive bands were identified using the Immun StarTM WesternCTM kit (Bio-Rad, Hercules, CA, USA), through the Molecular imager VersaDoc MP Systems (Bio-Rad, Hercules, CA, USA).

Quantity One software (v.4.6.6, Bio-Rad, Hercules, CA, USA) was used to quantify band intensities, these were referred to the loading control (actin). Experiments were performed in triplicate and data are expressed as means ± standard deviation (*n* = 3 ± SD).

### 4.4. TUNEL Assay and Quantitative Fragmented DNA Analysis

TdT-mediated dUTP nick-end labelling (TUNEL) (Promega, Madison, WI, USA, cod. G3250) was performed on whole-fixed embryos, as previously described by [26], in order to identify apoptotic nuclei. The fragmented DNA in apoptotic nuclei was observed by an Olympus BX50 fluorescence microscope, using a 20× objective.

Quantitative analysis of fragmented DNA was obtained by ImageJ 1.46r software (Bethesda, US-MD).

Experiments were performed in triplicate and data are expressed as means ± standard deviation (*n* = 3 ± SD).

### 4.5. Statistical Analysis

Quantitative data related to protein level and the TdT test from 3 independent experiments were used (*n* = 3 ± SD). A one-way analysis of variance (ANOVA) was used to compare the differences and the Tukey HSD test was used for post hoc testing. Data were checked for homogeneity of variance through the Levene’s test prior to analysis, to check that the assumptions of ANOVA were met. All statistical analyses were performed using the Statistica 13.2 software (StatSoft, Tulsa, OK, USA), and a *p*-value < 0.05 was considered significant.

## 5. Conclusions

Anthropogenic activities increase the rate of changes to the marine environment. Stress resistance mechanisms and phenotypic plasticity will be key factors to determine species survival [37,47].

The possibility to predict the consequences of a single pollutant, as V, for which an increase in its environmental concentration could be expected, is of considerable interest.

When facing stress, sea urchin embryos skillfully initiate several strategies. These could stimulate, in some cells, the cell cycle block (probably activating alternative pathway of damage repair). Here we show that, by exposing embryos to high V doses, they adopt an extreme strategy: cell-selective apoptosis. This seems a privileged tactic to protect the development plan, sacrificing just a few excessively injured cells.

In V-exposed embryos, alternative developmental phenotypes and adaptive responses are triggered, confirming that the success of embryogenesis depends on cellular mechanisms producing robustness to this life stage and proving once again the good capability of embryonic resilience under stress conditions [48].

These specific responses allowed us to elect the sea urchin embryo as being an adequate experimental animal model for investigations about cellular/molecular responses related to metals.

## Data Availability

Not applicable.
